# Calculating dose‐averaged linear energy transfer in an analytical treatment planning system for carbon‐ion radiotherapy

**DOI:** 10.1002/acm2.13866

**Published:** 2022-12-17

**Authors:** Weiwei Wang, Ping Li, Kambiz Shahnazi, Xiaodong Wu, Jingfang Zhao

**Affiliations:** ^1^ Department of Medical Physics Shanghai Proton and Heavy Ion Center Fudan University Cancer Hospital Shanghai Key Laboratory of Radiation Oncology (20dz2261000) Shanghai Engineering Research Center of Proton and Heavy Ion Radiation Therapy Shanghai China; ^2^ Institute of Modern Physics Applied Ion Beam Physics Laboratory Fudan University Shanghai China; ^3^ Department of Radiation Oncology Shanghai Proton and Heavy Ion Center Shanghai Key Laboratory of Radiation Oncology (20dz2261000) Shanghai Engineering Research Center of Proton and Heavy Ion Radiation Therapy Shanghai China; ^4^ Department of Medical Physics Shanghai Proton and Heavy Ion Center Fudan University Cancer Hospital Shanghai China

**Keywords:** carbon‐ion radiotherapy, dose averaged linear energy transfer (LETd), relative biological effectiveness (RBE)

## Abstract

**Background:**

Compelling evidence shows the association between the relative biological effectiveness (RBE) of carbon‐ion radiotherapy (CIRT) and the dose averaged linear energy transfer (*LETd*). However, the ability to calculate the *LETd* in commercially available treatment planning systems (TPS) is lacking.

**Purpose:**

This study aims to develop a method of calculating the *LETd* of CIRT plans that could be robustly carried out in RayStation (V10B, Raysearch, Sweden).

**Methods:**

The calculation used the fragment spectra in RayStation for the CIRT treatment planning. The dose‐weighted averaging procedure was supported by the microdosimetric kinetic model (MKM). The MKM‐based pencil beam dose engine (PBA, v4.2) for calculating RBE‐weighted doses was reformulated to become a *LET*‐weighted calculating engine. A separate module was then configured to inversely calculate the *LETd* from the absorbed dose of a plan and the associated fragment spectra. In this study, the ion and energy‐specific *LET* table in the *LETd* module was further matched with the values decoded from the baseline data of the Syngo TPS (V13C, Siemens, Germany). The *LETd* distributions of several monoenergetic and modulated beams were calculated and validated against the values derived from the Syngo TPS and the published data.

**Results:**

The differences in *LETds* of the monoenergetic beams between the new method and the traditional method were within 3% in the entrance and Bragg‐peak regions. However, a larger difference was observed in the distal region. The results of the modulated beams were in good agreement with the works from the published literature.

**Conclusions:**

The method presented herein reformulates the MKM dose engine in the RayStation TPS to inversely calculate *LETds*. The robustness and accuracy were demonstrated.

## 1 INTRODUCTION

Carbon‐ion beam is a high linear energy transfer (*LET*) radiation with lower distal doses than the heavier ions but higher *LET* than the lighter ions. The variable *LET* of carbon‐ion leads to variable RBEs. Therefore, CIRT requires an effective biophysical model for accurate quantification of RBEs throughout the entire range.^1^ Local effect model (LEM)^2^ and microdosimetric kinetic model (MKM)^2^ are currently the most frequently used models in clinical practice.

Although LEM and MKM use different approaches in calculating RBEs,^3^, ^4^, ^5^ the dose averaged *LET* (*LETd*), taking into account the primary ions and the light‐ion fragments, is the shared physical quality essential for determining the biological effectiveness.^6^, ^7^ Accordingly, ICRU‐93 recommends reporting the dose‐weighted *LET* for each treatment plan.^8^ *LETd* also has been acknowledged as an indicator strongly correlated to the clinical outcome in cancer radiotherapy.^9^, ^10^, ^11^

However, *LETd* calculation has not been widely available. Although dedicated Monte‐Carlo (MC) simulations have been used in some institutions,^12^, ^13^ accessing a fast MC engine for routine *LETd* calculation is naturally challenging for most of the particle therapy practitioners. Developing an accurate and easy *LETd* calculation method on commercially available treatment planning system (TPS) platforms would therefore be advantageous.

Herein, a *LETd* calculation method for CIRT plans was established based on the RayStation TPS (V10B, Raysearch, Sweden). The function of the MKM‐based pencil beam dose engine was reformulated from calculating RBE‐weighted doses to obtaining the *LETd*. The accuracy of the method was subsequently validated by comparing its results with the published data and with Syngo, the TPS used in the routine clinical practice of our institution.

## 2 METHODS AND MATERIALS

### 2.1 Procedural consideration

The kinetic energy is correlated with *LET*. Based on Equation (1), the *LETd* at the depth of *x* for a monoenergetic beam can be calculated:

(1) $$LETd\;\left(x\right)=\frac{\sum_{ij}d_{ij}\left(x\right)*LET_{ij}\left(x\right)*w_{ij}}{\sum_{ij}d_{ij}\left(x\right)*w_{ij}}\;$$

where *i* is ion type from Z = 1 to 6, *j* the kinetic energy of ion type i. $LET_{ij}\left(x\right)$, $w_{ij}$, and *d* donate ion/energy‐specific LET, relative weight, and the absorbed dose of the energy *j* of ion type *i*, respectively.

The RBE‐weighted dose $D_{RBE}\;$calculation in MKM is derived from the average number of lethal lesions in a nucleus, $L_{n}$, as calculated according to Equation (2):

(2) $$\begin{array}{ccc} L_{n} & = & \left(a_{0}+\beta *z_{1D}^{*}\right)*D_{Abs}+\beta *D_{Abs}^{2}\\ & = & -\ln S=a_{r}*D_{RBE}+\beta *D_{RBE}^{2} \end{array}$$

where $D_{Abs}$, $D_{RBE}$, and *S* denote the ion absorbed dose, RBE‐weighted dose, and the surviving fraction, *a*_0_ and $a_{r}$ are the linear‐quadratic (LQ) parameters of *LET* = 0 radiation and reference radiation. And β is independent of radiation types. The critical parameter $z_{1D}^{*}$ in Equation (2) is represented as:

(3) $$z_{1D}^{*}\left(x\right)=\frac{\sum_{i}d_{i}\left(x\right)*z_{1\mathrm{Di}}^{*}\left(x\right)*w_{i}}{\sum_{i}d_{i}\left(x\right)*w_{i}}$$

where $z_{1Di}^{*}\left(x\right)$, $w_{i}$, and $d_{i}\left(x\right)$ are $z_{1D}^{*}$, the relative weight, and the absorbed dose of the *i*th beam.

Subsequently,

(4) $$\;D_{RBE}=\frac{\sqrt{{\alpha_{r}}^{2}+4\beta L_{n}}-\alpha_{r}}{2\beta }\;$$

The objective here is to use the computational procedure of $z_{1D}^{*}\left(x\right)$ of Equation (3) in the TPS to obtain LETd(x). This amounts to substituting the term $z_{1Di}^{*}\left(x\right)$ with $LET_{ij}\left(x\right)$. With this substitution, the computation of $D_{RBE}\;$would result in a fatigious quantity denoted as *ξ_LET_*,

(5) $$\begin{array}{ccc} & & \left(a_{0}+\beta *LETd\left(x\right)\right)*D_{Abs}+\beta *D_{Abs}^{2}\\ & & \;=a_{r}*\xi_{LET}+\beta *{\xi_{LET}}^{2}, \end{array}$$

from which the LETd(x) can be recovered by the following:

(6) $$\begin{array}{ccc} LETd\;(x) & = & \left[\frac{a_{r}*\xi_{LET}+\beta *\left({\xi_{LET}}^{2}-D_{Abs}^{2}\right)}{D_{Abs}}-a_{0}\right]\\ & & *\;\beta^{-1}. \end{array}$$

### 2.2 TPS configuration and calculation

To implement the afore‐described concept and technique, an independent non‐clinical module, “***LETd module*** *”*, was created in the RayStation TPS for the sole purpose of calculating *LETds*. In this module, the $z_{1Di}^{*}\left(x\right)$ was replaced by $LET_{ij}\left(x\right)$, as such, the output of the RBE‐weighted dose based on the analytic pencil beam algorithm (PBA, v4.2) would yield the factitious parameter *ξ_LET_* as described by Equation (5).

A two‐step approach was performed for calculating the *LETd* for a given treatment plan: (i) the ***LETd module*** was applied to calculate the *ξ_LET_* based on the optimized $D_{Abs}$; (ii) the *LETd* was computed by Equation (6). Consistent with the algorithm used in the TPS, the calculation of *LETd* assumes water equivalence.

### 2.3 Validations

The Syngo system has been our primary TPS since the beginning of clinical operation in 2014, and was used to validate the proposed *LETd* inverse calculation method. Since the Syngo system does not provide *LETd* directly as part of the treatment plan output, an in‐house software was developed to calculate the *LETds* of the Syngo plans using Equation (1) with the fragment spectra read out from the Syngo plans and the $LET_{ij}\left(x\right)$ in the Syngo beam data library.

To further consolidate the baseline beam data between the Syngo and RayStation for comparison purposes, the fragment‐specific (Z = 1–6) *LETds* of a 418.74 MeV/u monoenergetic beam were generated from both Syngo and RayStation, and a scaling factor was introduced for each ion type. The $LET_{ij}\left(x\right)$ in the RayStation beam data library was subsequently adjusted by applying these scaling factors.

#### 2.3.1 Monoenergetic beams

Three monoenergetic beams with 149.96, 258.75, and 348.51 MeV/u and a 3 mm ripple filter were used to generate dose distributions of 10 × 10 cm^2^ field. The *LETds* from the Syngo plans were calculated using the in‐house software and subsequently compared to the *LETds* from the RayStation plans using the new inverse method. The step sizes in the Bragg peak regions of the Syngo‐*LETds* and the RayStation‐*LETds* were < 1.0 mm and constantly 1.0 mm, respectively. The local differences between the two sets of *LETds* were calculated. Due to the steep gradient in the Bragg peak region, the averaged *LETd* values in 2.0 mm interval were used to compute the local differences.

#### 2.3.2 Modulated beams

Kanematsu et al. provided a fitting‐based approach for calculating the *LETds* of two SOBP plans of 350 MeV/u.^13^ These plans used the passive scattering delivery technique,^14^ one with a single beam while the other with two opposing beams. Kanematsu et al. also presented the corresponding MC‐based *LETd* distributions from Kanai et al.^15^ In this study, we generated the modulated plans using the same beam parameters of Inaniwa et al.’s study and then calculated the corresponding *LETds*, to evaluate the accuracy of our method.

## 3 RESULTS

### 3.1 Beam data matching

**FIGURE 1:**
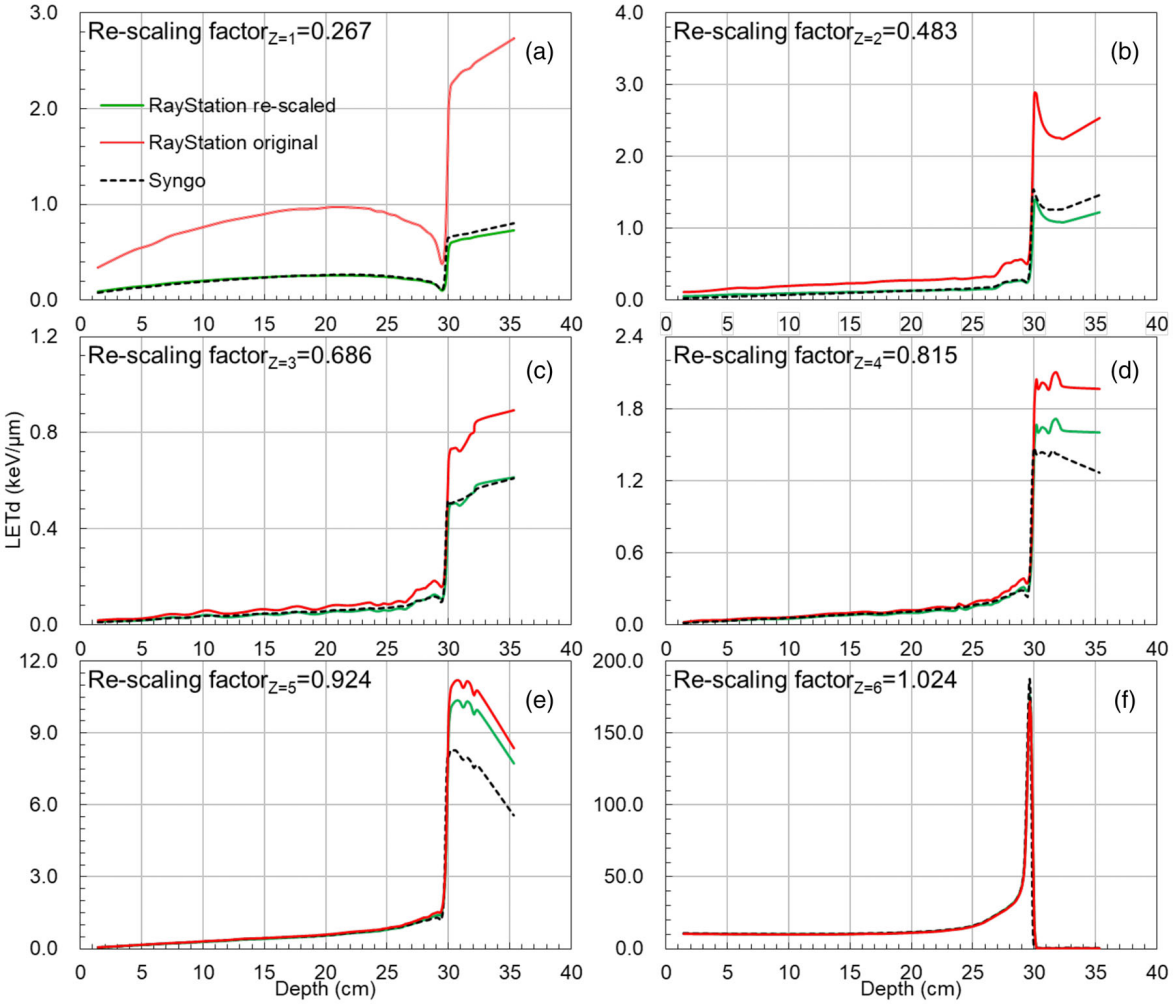
The *LETd* components of Z = 1 (a), 2 (b), 3 (c), 4 (d), 5 (e), and 6 (f) of 418.74 MeV/u. The black dash lines are the Syngo‐*LETds*, while the green and red solid lines are the re‐scaled and original RayStation‐*LETds*

Figure 1 shows the ion‐specific *LETd* components, that is, Z = 1 (a), 2 (b), 3 (c), 4 (d), 5 (e), and 6 (f), of the 418.74 MeV/u beams from the Syngo and the RayStation with the re‐scaling factors applied. The re‐scaling factors were 0.267, 0.483, 0.686, 0.815, 0.924, and 1.024 for the ion types Z = 1–6.

### 3.2 Validation using monoenergetic beams

**FIGURE 2:**
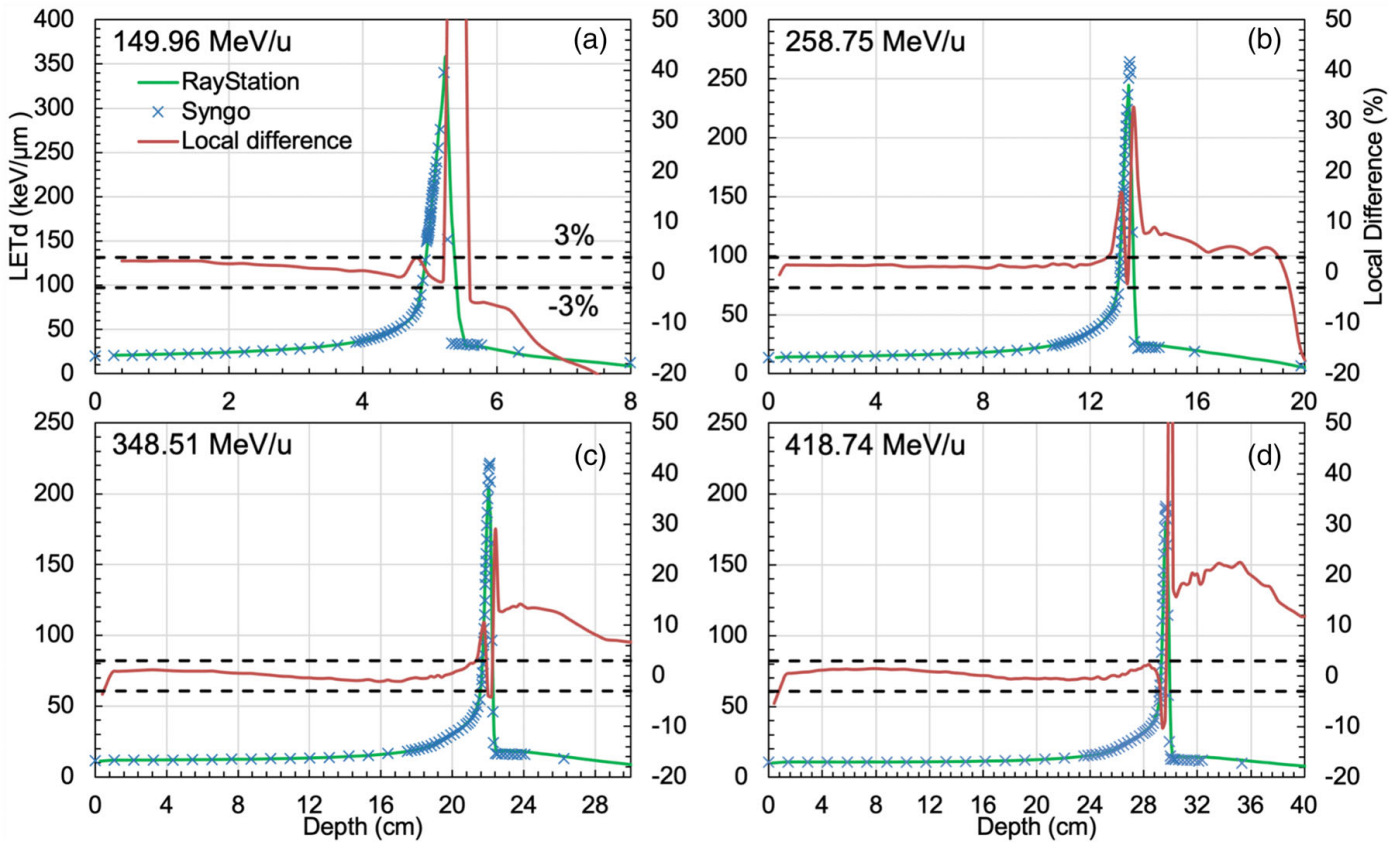
The *LETds* for monoenergetic beams of 149.96 (A), 258.75 (B), 348.51 (C), and 418.74 (D) MeV/u. The crosshairs represent the Syngo‐*LETds*. The green lines denote the RayStation‐*LETds*. The brown lines indicate the local difference

The *LETds* of 149.96 (a), 258.75 (b), 348.51 (c), and 418.74 (d) MeV/u are shown in Figure 2, with the difference (%) between the two methods of calculation overlaid.

### 3.3 Validation using modulated beams

**FIGURE 3:**
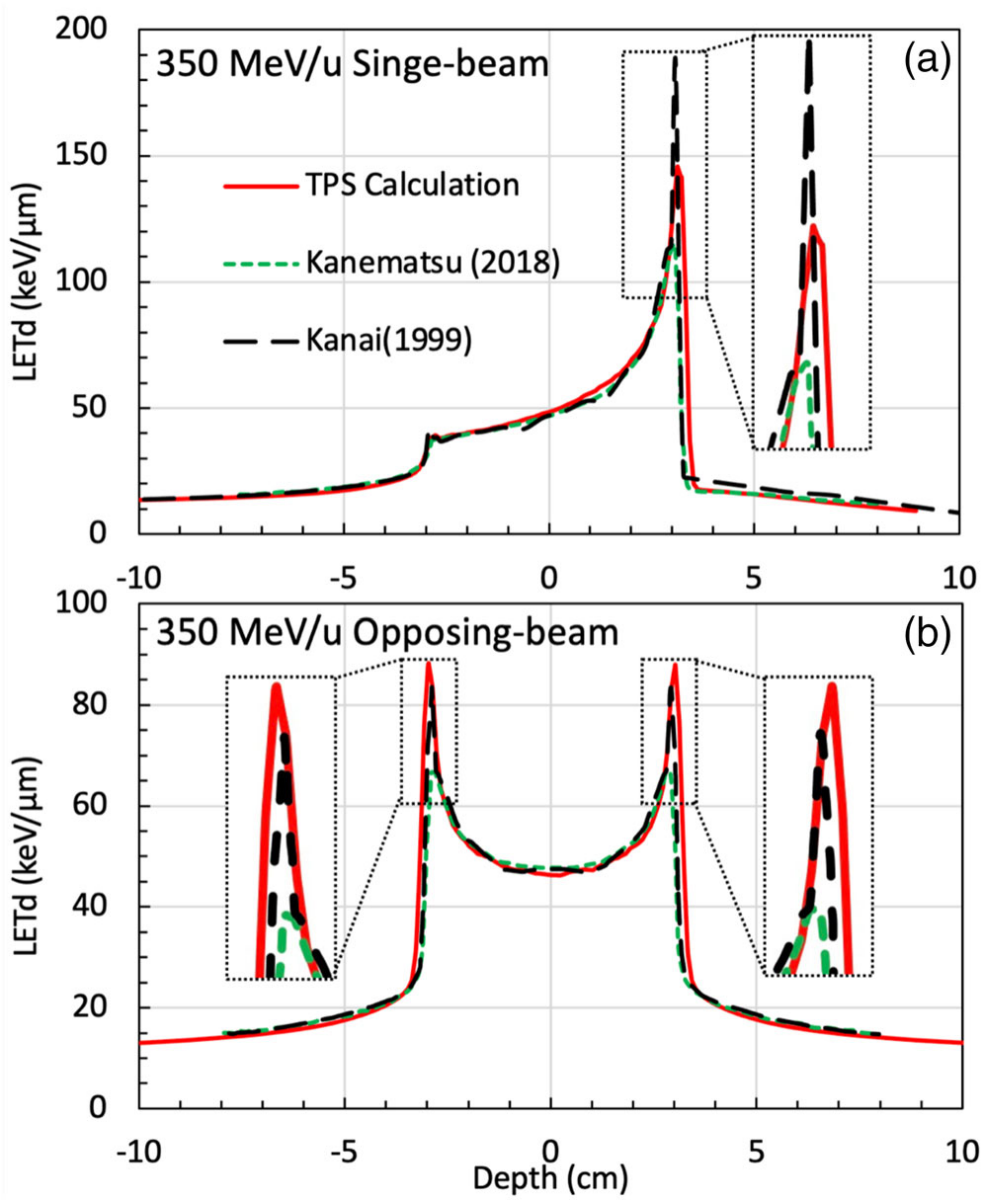
The *LETd* distributions of 350 MeV/u SOBP plans. The red and solid lines were from our study; the green dash lines and black dash lines are from Kanematsu et al. and from Kanai et al. “a” and “b” are the *LETds* of single‐beam and opposing‐beam plans

Figure 3 illustrates the results of our new method (red and solid lines), the corresponding results from the fittings of Kanematsu et al. (green dash lines), and the MC results from Kanai et al. (black dash lines). In Figure 3, “a” and “b” are the *LETd* distributions for the single‐beam and opposing‐beam cases. The results from our study are in close agreement with the previous work by Kanai et al. The sub‐millimetre difference in range reflects the slight difference in the beam qualities.

## 4 DISCUSSIONS

An inverse approach for calculating the *LETd* using the RayStation TPS for CIRT plans was presented herein. This method can be implemented by creating a separate module using the same fragment spectra and the PBA dose engine in RayStation TPS. We employed this approach to calculate the *LETds* of several monoenergetic beams and modulated beams and compared them to the results obtained from the Syngo TPS and with MC calculations.

One of the benefits of CIRT is the higher RBE, attributed to its higher *LETd*. *In‐vivo* studies show that high *LETd* can help overcome the radio‐insensitive tumours under hypoxic conditions.^4^, ^16^, ^17^ Furthermore, several recent studies have demonstrated the potential association of *LETd* of CIRT with tumour recurrence or normal tissue toxicities.^9^, ^10^, ^11^ These suggest that clinical outcomes could be further improved by incorporating *LETd* in the planning optimization process. Although *LETd* can be derived through MC simulation,^18^ measurements,^12^ or analytic calculations,^19^ evaluation or optimization of LETd in patients’ treatment plans remains challenging. The first step towards this endeavour would be to make *LETd* distribution of a dose plan easily accessible. The described method allows the RayStation users to gain easy access to the *LETd* distribution of their plans and may allow further studies to correlate the clinical response with *LETd* in CIRT.

The currently available *LETd* calculations such as FRoG,^18^ although quite powerful, requires additional beam commissioning, in particular, feeding a pencil beam dose engine with the Pre‐MC‐derived *LETds* of different energies. Our approach utilizes the existing RBE‐weighted dose engine without the need for additional commissioning data.

The sub‐millimetre range deviations in Figure 2 are likely caused by interpolation of the beam parameters of arbitral energy from a set of discrete beam energies and fragment spectra. Furthermore, the *LETd* showed larger deviations in the tail of the *LETds* in depths. This can be explained by the fragment deviations of the ion types Z = 4 and 5 as shown in Figure 1.

Using PBA to inversely calculate the *LETd* with the presented algorithm inherently lacks the sensitivity to tissue variability and inhomogeneity (i.e., lung tissue), which should be taken into consideration when used in clinical settings.

## 5 CONCLUSIONS

A novel method of calculating *LETd* for CIRT plans was described. The accuracy and robustness were demonstrated. The process is based on the fragment spectra and PBA in the RayStation TPS, which could be easily implemented in any CIRT centre using RayStation TPS.

## AUTHOR CONTRIBUTIONS

Jingfang Zhao and Xiaodong Wu designed the study. Weiwei Wu performed the study. Kambiz Shahnazi and Ping Li helped analysed the results. Weiwei Wu and Jingfang Zhao wrote the manuscript. All authors reviewed and approved the manuscript.

## CONFLICT OF INTEREST

The authors declare no conflict of interest.
